# Impact of Abiotic Stress on Rice and the Role of DNA Methylation in Stress Response Mechanisms

**DOI:** 10.3390/plants13192700

**Published:** 2024-09-26

**Authors:** Ming Yin, Shanwen Wang, Yanfang Wang, Ronghua Wei, Yawei Liang, Liying Zuo, Mingyue Huo, Zekai Huang, Jie Lang, Xiuqin Zhao, Fan Zhang, Jianlong Xu, Binying Fu, Zichao Li, Wensheng Wang

**Affiliations:** 1 State Key Laboratory of Crop Gene Resources and Breeding, Institute of Crop Sciences, Chinese Academy of Agricultural Sciences, Zhong-Guan-Cun South Street 12#, Beijing 100081, China; 82101219104@caas.cn (M.Y.); w202219951228@163.com (Y.W.); lyawei999@gmail.com (Y.L.); z1983212403@163.com (L.Z.); 18331273792@163.com (M.H.); zhaoxiuqin@caas.cn (X.Z.); zhangfan03@caas.cn (F.Z.); xujianlong@caas.cn (J.X.); fubinying@caas.cn (B.F.); 2 Frontiers Science Center for Molecular Design Breeding, Key Laboratory of Crop Heterosis and Utilization (MOE), Beijing Key Laboratory of Crop Genetic Improvement, College of Agronomy and Biotechnology, China Agricultural University, Beijing 100193, China; 3 Southwest United Graduate School, Kunming 650092, China; shanwen.wang@foxmail.com; 4 Center of Innovation for Perennial Rice Technology in Yunnan, School of Agriculture, Yunnan University, Kunming 650091, China; hzk1@mail.ynu.edu.cn (Z.H.); jielang@mail.ynu.edu.cn (J.L.); 5 National Nanfan Research Institute (Sanya), Chinese Academy of Agricultural Sciences, Sanya 572024, China; 6 Department of Agronomy, Hebei Agricultural University, Baoding 071001, China; weironghua1999@163.com

**Keywords:** DNA methylation, abiotic stresses, epigenetic, rice, tolerance

## Abstract

With the intensification of global climate change and the increasing complexity of agricultural environments, the improvement of rice stress tolerance is an important focus of current breeding research. This review summarizes the current knowledge on the impact of various abiotic stresses on rice and the associated epigenetic responses (DNA methylation). Abiotic stress factors, including high temperature, drought, cold, heavy metal pollution, and high salinity, have a negative impact on crop productivity. Epigenetic changes are key regulatory factors in plant stress responses, and DNA methylation is one of the earliest discovered and thoroughly studied mechanisms in these epigenetic regulatory mechanisms. The normal growth of rice is highly dependent on the environment, and changes in the environment can lead to rice sterility and severe yield loss. Changes in the regulation of the DNA methylation pathway are involved in rice’s response to stress. Various DNA methylation-regulating protein complexes that function during rice development have been identified. Significant changes in DNA methylation occur in numerous stress-responsive genes, particularly those in the abscisic acid signaling pathway. These findings underscore the complex mechanisms of the abiotic stress response in rice. We propose the effective improvement of tolerance traits by regulating the epigenetic status of rice and emphasize the role of DNA methylation in abiotic stress tolerance, thereby addressing global climate change and ensuring food security.

## 1. Introduction

Rice (*Oryza sativa* L.) is among the three major food crops in the world and a staple food for 3.2 billion people [1]. The United Nations predicted that the world’s population will grow from 6.2 billion in 2000 to 9.5 billion in 2050 [2]. With the steady growth of the population, the demand for food production is escalating. Furthermore, rice production is confronted with the challenge of yield diminution attributable to diverse abiotic stress factors in addition to population growth [3]. Therefore, understanding the mechanisms of rice tolerance to abiotic stresses and the development of stress-tolerant varieties has become a critical research focus to ensure food security.

Since the nineteenth century, the global warming phenomenon has been exacerbated by greenhouse gas emissions arising from human activities worldwide, ultimately resulting in an elevated likelihood of extreme weather events [4]. Extreme weather imposes drought stress [5], temperature stress, heat stress [6], and cold stress [7]) on rice. As a consequence of urban and industrial development, rice crops also suffer from salt stress [8] and heavy metal stress [9]. Rice has evolved mechanisms to survive under constantly changing extreme environmental conditions [9,10,11,12,13,14]. However, given that the tolerance to abiotic stress is a trait controlled by multiple genes, the elucidation of the relevant mechanism is challenging [11]. With the progress in research into molecular regulatory mechanisms, a growing body of evidence indicates that epigenetic regulation plays an important role in rice stress tolerance [15,16]. Among epigenetic regulatory mechanisms, DNA methylation occupies a pivotal position in conferring stress tolerance in rice [17,18]. DNA methylation is crucial for the adaptive response of rice to salinity stress, allowing the plants to maintain ionic homeostasis and survive in a saline environment. For example, Xia et al. [19] reported that differences in methylation were associated with osmotic tolerance in rice seedlings. Wang et al. [20] determined that salt stress induces a decrease in DNA methylation, specifically in roots at the seedling stage. DNA methylation plays an essential role in the resilience of rice to extreme temperature stress and in enhancing tolerance. Folsom et al. [21] observed that DNA methylation is correlated with seed size under heat stress. In addition, DNA methylation improves the tolerance of rice to heavy metal pollution, protecting the plant from harmful toxicity effects. Cong et al. [22] observed that DNA hypomethylation-associated transcriptional rewiring enables resistance to heavy metal (mercury) stress in rice.

DNA methylation is crucial for rice’s adaptive response to both salinity and extreme temperature stresses, as it maintains ionic homeostasis and enhances tolerance capabilities, thereby supporting survival in challenging environments. In addition, DNA methylation improves the tolerance of rice to heavy metal pollution, protecting the plant from harmful toxicity effects. Thus, DNA methylation is immensely important in the stress response of rice, enabling the plant to cope with diverse abiotic stressors effectively. Elucidation of the underlying mechanisms of DNA methylation will facilitate the development of stress-tolerant rice varieties, thereby contributing to future food security.

## 2. Impacts of Abiotic Stress Factors on Rice

**Figure 1:**
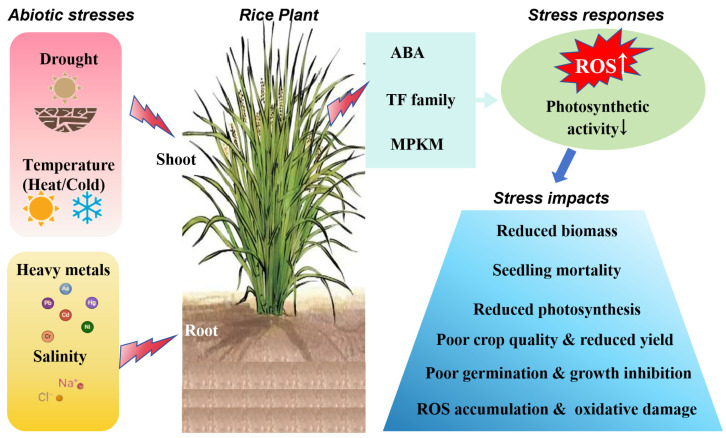
Schematic diagram of the mechanism of abiotic stress response in rice.

Abiotic stress poses a serious environmental threat, notably presenting a substantial risk to crop production. Among the various types of abiotic stress, drought stress, temperature stress, salt stress, and heavy metal stress have particularly profound impacts on rice production [23] (Figure 1).

### 2.1. Drought Stress

It is estimated that, globally, drought impacts approximately 23 million ha of rice production, potentially leading to a reduction of 1.8 billion kg in rice output annually [24]. The reduction in available water in the soil as a result of insufficient precipitation is the main reason that crops experience drought stress. In addition to precipitation, transpiration, the water-holding capacity of the soil, and the water demand of crops are the principal factors affecting crop drought [25].

When plants lose water, the intracellular osmotic pressure increases, which significantly affects cell expansion and energy synthesis and causes substantial oxidative damage [26,27,28]. The plant water deficit triggers signals through several major and minor signaling pathways, primarily the mitogen-activated protein kinase (MAPK) pathway, the sucrose non-fermenting-1-related protein kinase (SnRK) pathway, and the abscisic acid (ABA) signal transduction pathway [29,30]. Yu et al. [31] proposed the hypothesis that osmotic stress signals may comprise a mixture of multiple physiological signals. Drought stress mainly affects “protective membrane” genes that control water and ion uptake/transport, and regulatory genes for signaling/transcriptional control in crops. Plant drought stress signals are transmitted from the plasma membrane to the nucleus via MAPKs [32] and receptor-like kinases (RLKs) [33]. Subsequently, a drought tolerance regulatory network is activated, consisting primarily of hormonal signals and other metabolites, including reactive oxygen species (ROS), proteins, and other osmotic stress catabolites [34].

Under drought stress, rice initially produces large amounts of ABA, which reduces stomatal conductance and transpiration water loss [35]. Numerous MYB and MYC transcription factors are involved in ABA synthesis [36]. The overexpression of *OsMYBR1* [37] and *OsMYB48-1* [38] affect ABA synthesis in rice and enhance drought stress tolerance. In addition to ABA production, an additional major phytohormone produced under drought stress is auxin [39]. For example, the auxin-related genes *OsPIN3t* [40] and *OsGH3-2* [41] affect crown root formation at the seedling stage, and their overexpression significantly improves drought tolerance in rice. In addition to phytohormones, a multitude of sugars, amino acids, ROS, and antioxidant substances accumulate in large quantities. When a water deficit is sensed, the plant starts to produce and store large amounts of sugars and uses these sugars to supply energy during the drought stress phase [42,43]. Glucose, fructose, galactose, and sucrose synthesis are elevated significantly under drought stress [43,44]. Subsequently, numerous amino acids are produced in response to drought stress; their primary role is to increase the water retention capacity of plant cells and reduce ROS accumulation [45]. Such amino acids include proline, lysine, threonine, methionine, tryptophan, and phenylalanine [46,47,48]. Furthermore, rice produces large amounts of ROS and antioxidant substances under drought stress, of which ROS is a major cause of oxidative damage but also are important stress signaling molecules [49]. In response to oxidative damage, plants produce diverse antioxidant substances, of which the most important are flavonoids, such as anthocyanins and flavonols [50], and antioxidant enzymes, such as peroxidase (POD) and superoxide dismutase (SOD) [51].

### 2.2. Temperature Stress

Among the various environmental stresses, temperature stress has a significant effect on the yield of rice. More than 1.5 million ha of rice production worldwide is significantly affected by temperature stress each year [52]. All plants have an optimal temperature range for growth. Rice also has its optimal growth conditions. With the current frequency of extreme weather events, a focus for optimal rice growth is on temperature tolerance. For example, the maximum temperature for rice growth should not exceed 35 °C, and the minimum should not be lower than 17 °C. Thus, temperature stress can be divided into cold stress and heat stress. Under heat stress, rice yield is predicted to decline by 8% for every 1 °C increase in global average temperature. Under cold stress, the reduction in rice yields can be as much as 87% [53].

When the external environmental temperature changes, the permeability of the plasma membrane of plant cells is affected initially, which subsequently leads to remodeling of the cell wall structure and changes in cell membrane fluidity [54]. Low temperature causes obvious external damage to rice, such as low germination rates, stunted growth and abnormal development of seedlings or even death, and low percentage seed setting. Cold stress stimulates a series of physiological and metabolic changes in rice, such as changes in electrolyte leakage, sucrose, lipid peroxides, proline, and other metabolites, in addition to ROS, malondialdehyde, and certain endogenous phytohormones, such as ABA and gibberellins [55].

Under cold stress, rice senses cold signals mainly through the *CHILLING TOLERANCE DIVERGENCE 1* (*COLD1*) and *DAIKOKU DWARF (Rice G-protein α subunit 1, RGA1)* receptors, which are located on the cytoplasmic and endoplasmic reticulum membranes, respectively [56]. There are distinct differences in the cold sensor encoding gene *COLD1* between *indica* and *japonica* rice. A single nucleotide difference in *COLD1* can significantly change the cold tolerance of rice. The vitamin E–vitamin K1 subnetwork is a downstream pathway of *COLD1* and is the core regulatory node that determines the difference in cold tolerance between *indica* and *japonica* rice. In contrast, rice shows morphological and physiological symptoms under high-temperature stress. The morphological symptoms mainly include seedling death, decreased tillering, decreased percentage seed setting, increased grain chalkiness, and decreased grain quality. Physiological symptoms primarily include membrane damage, excessive accumulation of ROS, obstruction of photosynthesis, disordered carbohydrate metabolism and distribution, and imbalance in phytohormone concentrations.

The molecular mechanisms involved in response to heat stress in rice include cell wall structure remodeling, changes in cell membrane fluidity, changes in cell membrane-localized Ca^2+^ channel-mediated Ca^2+^ concentrations, and changes in cell membrane-localized respiratory burst oxidase homolog-mediated ROS concentrations as early events in response to heat stress [13]. An increase in Ca^2+^ and ROS concentrations in the cytoplasm stimulates a series of downstream heat-stress-response pathways. Among the pathways, the transcriptional regulatory network centered on the heat shock transcription factor *HSFA1* is essential for plants to respond to heat stress. In addition, protein homeostasis under heat stress is particularly important for rice to cope with high temperatures and is influenced by factors including endoplasmic reticulum-dependent protein quality control, effective clearance of toxic proteins, and the translational regulation level [57].

Photoreceptor red light-receptor phytochrome B (phyB) is a thermosensor, and temperature changes affect its far-red-light-absorbing form (Pfr) to red-light-absorbing form (Pr) reversion rate. The common receptor phyB distinguishes light and temperature signals through conformational changes and phase separation, respectively [58]. More specific response systems to heat stress include the unfolded protein response induced by disturbances in protein homeostasis, the ubiquitin–26S proteasome system influenced by normal RNA function, and translational regulation to maintain protein homeostasis [59]. In Arabidopsis, the 26S proteasome subunit RPT2a promotes post-transcriptional gene silencing (PTGS) through the SUPPRESSOR OF GENE SILENCING 3 (SGS3)/RNA DEPENDENT RNA POLYMERASE 6 (RDR6) pathway. This pathway functions to convert single-stranded RNAs to double-stranded RNA, which affects RNA quality to control foreign RNAs [60]. The PTGS mechanism functions through short interfering RNAs (siRNAs) and microRNAs (miRNAs). SGS3 is a dsRNA-binding protein that plays an important role in regulating the small RNA pathway. Furthermore, rice produces numerous antioxidant substances and enzymes, such as POD, under temperature stress [61].

Both cold and heat stresses are sensed by plasma membrane proteins, such as calcium channel proteins, and lead to Ca^2+^ inflow. This is followed by activation of calcium-responsive protein kinases (creatine phosphokinases; CPKs), calcium/calmodulin-regulated receptor-like kinase (CRLK1), and MAPK, among other proteins, which ultimately regulate the expression of downstream cold and heat response genes [62]. Eventually, a regulatory network mainly regulated by hormonal signals is activated, acting in combination with other metabolites or proteins (malondialdehyde, guanosine triphosphate kinase, alglucan D-trehalose anhydrous [under cold stress], and heat shock proteins [under heat stress]) [59,63]. Plants exposed to temperature stress mainly regulate the expression of metabolic enzymes and associated genes to trigger the expression of a multitude of transcription factors, thus activating the expression of many downstream temperature stress tolerance genes [64].

### 2.3. Salt Stress

Salt stress is an important abiotic stress that increasingly affects crop growth and development and is an important constraint on the improvement in rice yields. It is estimated that the annual reduction in food production caused by salt stress accounts for 20% of global food production [65].

Salt stress causes plants to absorb large quantities of Na^+^ and Cl^−^, leading to osmotic stress and ionic stress [66]. High osmotic pressure causes water loss, which induces the leaf stomata to close and limits photosynthesis, thus affecting the normal growth and metabolism of the plant. The plant then reduces the cytoplasmic water potential by accumulating osmoprotectants, which are used to protect the plant from salt damage [67]. Under high salinity, the Ca^2+^ concentration rises rapidly. Calcium ions are an early response signal to salt, are translocated intracellularly, and are involved in regulating calmodulin, calcium-dependent protein kinases (CPKs), and other proteins to induce Na^+^ efflux from the cell to achieve ion homeostasis. The osmoprotectant will accumulate in large amounts and will reduce the cellular water potential, thus enhancing plant tolerance to salt stress.

ABA is the first to accumulate under salt stress and later binds to the PYRABACTIN RESISTANCE 1-LIKE (PYL) protein, which then interacts with PROTEIN PHOSPHATASE 2C (members of the type 2C protein phosphatase; PP2C) to form the PYL–ABA–PP2C complex, which promotes the production of SNF1-related protein kinases (SnRK2) and in turn maintains ion homeostasis, scavenges ROS, and promotes plant adaptation to high-salt stress [51]. Ethylene is an additional important class of hormones in the response to salt stress in rice. For example, when ethylene production is elevated, expression of the ethylene signaling response genes *MHZ6/OsEIL1* and *OsEIL2* are upregulated, and K^+^ transporter protein expression is suppressed, thus increasing the sensitivity of rice to salt [68]. Ethylene can also regulate salt tolerance in rice through ROS homeostasis [69]. In addition to phytohormones, plants synthesize organic substances of high molecular weight, such as sugars and alcohols, which increase the osmotic potential of cells and enhance their ability to absorb water in a hyperosmotic environment [70]. In addition to the synthesis of organic matter, plants enrich K^+^ from the environment through transporter proteins. Plants transfer Na^+^ to the vesicles by regulating high-affinity K transporters (HKTs) and promote the efflux of Na^+^ to maintain normal metabolic activity in cells [71]. Eventually, ROS are eliminated through multiple enzyme systems, but rice produces these enzymes with less energy consumption, probably because of the genes associated with the enzymes or energy-related genes [72].

### 2.4. Heavy Metal Stress

In recent years, owing to industrial development and widespread fertilizer application, heavy metal pollution in farmland has become a major environmental problem [73]. Approximately 235 million ha of agricultural soil are contaminated with heavy metals worldwide, predominantly in countries such as Japan, China, India, and Australia [74]. Large amounts of heavy metal elements, such as cadmium (Cd), arsenic (As), lead (Pb), and copper (Cu), have gradually accumulated in cultivated land and damage the growth of crop plants, and in turn affect humans through the food chain [75]. In China, according to data published by the Ministry of Environmental Protection in 2014, Cd is the most frequent primary pollutant, exceeding the maximum allowable concentration in soil at 7% of sites [76].

Cadmium is highly mobile and is easily absorbed by rice plants, resulting in the production of large amounts of rice grains contaminated with Cd in China [77]. Rice adsorbs soil Cd ions mainly through other ion channels, such as the manganese (Mn) ion transporter *LOW CADMIUM ACCUMULATION 1* (*LCD1*, *OsNRAMP5*) and the iron (Fe) ion transporters *IRON-REGULATED TRANSPORTER 1* (*OsIRT1*) and *OsIRT2*, whereas no root-specific Cd-uptake transporter proteins have been identified in rice [78]. After adsorption by the root system, Cd ions are enriched in the roots, hindering the proliferation of root cells and affecting the growth of the root system [79]. Upon its transport from the roots to the stems and leaves, Cd affects photosynthesis and enzyme activities, and accumulation to phytotoxic concentrations causes cellular damage and production of high concentrations of ROS [80]. Under Cd stress, rice plants show yellowing of the stems and leaves, leaf abscission, and a significant decrease in plant dry matter [81].

Rice plants principally use the following strategies to cope with Cd stress. The first strategy is regionalization, which is achieved by transporting Cd ions to plant organs or subcellular regions with lower metabolic activity, thus achieving detoxification [82]. For example, Cd is stored in vesicles to reduce the effect of Cd on the cytoplasmic matrix with other cell organelles [83]. The second coping mechanism is Cd chelation and translocation, whereby the plant uses phytochelatins and metallothioneins to bind Cd to prevent its diffusion in the cells [84]. Subsequently, metal transporter proteins, such as members of the yellow stripe-like protein family, ZRT and IRT-like protein family, and copper transport protein family, are used to transport Cd to the extracellular compartment [85]. The third strategy is the production of phytohormones, flavonoids, antioxidant enzymes, and other antioxidant substances, such as salicylic acid (SA), ABA, POD, SOD, and other metabolites, to reduce the severity of oxidative damage caused by heavy metal accumulation and to promote normal development [86].

Lead toxicity mainly disturbs rice growth processes (such as seed germination, root elongation, seedling development, plant growth, transpiration, water and protein content, and photosynthesis activity) and reproductive development through lipid peroxidation. Lead toxicity causes inhibition of ATP production, lipid peroxidation, and DNA damage through the overproduction of ROS. Lead can have negative effects on all stages of rice vegetative growth, e.g., by altering chloroplast ultrastructure, hindering the electron transport chain, inhibiting tricarboxylic acid (TCA) cycle enzymes, impairing the absorption capacity for essential elements such as magnesium and Fe, and disrupting stomatal opening and closing resulting in CO_2_ deficiency in vivo. However, rice plants can detoxify Pb through various strategies, including reducing the uptake of Pb into the cell, sequestering Pb in vacuoles in the form of complexes, and binding of Pb by phytochelatins, glutathione, and various amino acids. In addition, the activation of various antioxidants to combat the increased production of Pb-induced ROS constitutes a secondary defense system [82].

Copper stress can cause diverse physiological and biochemical changes in rice, such as root damage, nutrient deficiency, and photosynthesis inhibition. Copper toxicity is first manifested in the roots and is then exerted in the shoots, where it affects various physiological processes. High concentrations of Cu in the soil can inhibit and impair root growth, resulting in reduced nutrient and water uptake. Root growth inhibition is often associated with rupture of the root epidermis and outer cortex. Copper ions may regulate phytohormones, such as melatonin, auxin, and ABA, in rice root cells to change the proliferation rate of root meristems, thereby affecting root development. Given the antagonism between metal ions, Cu ion stress can inhibit the uptake of phosphorus and potassium in rice. In addition, excessive Cu in the soil disturbs the absorption and homeostasis of essential metals, including Fe, Mn, and zinc (Zn), resulting in stunted rice root growth, thus greatly reducing plant productivity and yield [87]. Excessive Cu may modify the chloroplast ultrastructure and composition of photosynthetic membranes, cause oxidative stress in plant cells, reduce the contents of photosynthetic pigments and electron carriers, and hinder photosynthetic electron transport. Cao et al. [88] observed that, under Cu ion stress, several intermediates in the TCA cycle were downregulated, including succinic acid (−2.06-fold), malic acid (−4.74-fold), citric acid (−5.02-fold), and aconitate (−3.05-fold), indicating that carbohydrate metabolism was severely disturbed. The downregulation of intermediate metabolites in the TCA cycle, purine metabolism, and sucrose metabolism pathways may be at least partly responsible for the inhibition of rice growth under Cu toxicity.

## 3. DNA Methylation Regulatory Mechanisms of Rice in Response to Abiotic Stress

As genetic studies have intensified, numerous epigenetic mechanisms associated with plant responses to abiotic stress have been reported [15]. Epigenetic modifications of the rice genome mainly include genome-wide DNA methylation, RNA methylation, histone modification, chromatin remodeling, non-coding RNA-mediated gene expression regulation, and nucleosome positioning. These modifications reveal the complex mechanisms of epigenetic regulation, which ultimately affect the expression of developmental genes [89]. DNA methylation is affected by the addition (or removal) of methyl groups to CG/CHG/CHH (where H is an A, C, or T residue) sites through the activity of DNA (de-)methylation enzymes [90,91]. DNA methylation involves the enzymatic transfer of the methyl group (CH3-) from the *S*-adenosylmethionine molecule to the cytosine residue of specific DNA sequences, resulting in the formation of methylated nucleobases, such as 5-methylcytosine (5mC), N6-methyladenine, and 7-methylguanine, in rice. Among the various types of methylation, 5mC is one of the most deeply studied epigenetic regulatory mechanisms in rice. The type of DNA methylation discussed in this paper, if not specifically noted, is 5mC. This process represents a crucial epigenetic modification that regulates gene expression and maintains genome stability. In higher plants, the entire methylation of DNA is divided into de novo methylation, maintenance methylation, and demethylation [92,93,94]. Changes in the methylation level of DNA affect transposon activity, gene expression, and genomic stability [92].

**Figure 2:**
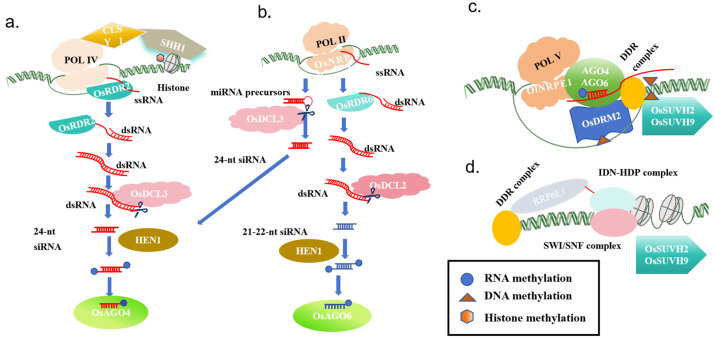
RNA-directed DNA methylation pathway model (**a**) POL IV-dependent siRNA biogenesis; (**b**) POL II-dependent siRNA biogenesis; (**c**) POL V-mediated de novo and maintenance methylation; (**d**) chromatin alterations.

In plants, de novo methylation is mediated by the RNA-directed DNA methylation (RdDM) pathway [95] (Figure 2). The plant-specific RdDM pathway involves complex interactions between various RNAs and proteins [96,97]. It incorporates a series of biological macromolecules composed of small interfering RNAs (siRNAs), scaffold RNAs, and numerous protein molecules. It is the only pathway in plants that can achieve de novo methylation of previously unmethylated DNA regions [98,99]. Two plant-specific factors are central to the mechanism of the RdDM pathway—POL IV and Pol V, both of which are RNA polymerase II (POL II)—related RNA polymerases [100,101]. Based on current models, the canonical RdDM pathway encompasses two successive stages: (a) biogenesis of 24-nucleotide (nt) siRNAs, which requires POL IV, RDR2, and DCL3, and (b) the initiation of de novo methylation, which depends on scaffold RNAs produced by POL V, 24-nt siRNAs bound to AGO4, and the de novo DNA methyltransferase DOMAINS REARRANGED METHYLTRANSFERASE 2 (DRM2) [100]. The RdDM pathway generates single-stranded RNA and double-stranded RNA, which are cleaved into 24-nt sRNAs by DICER-LIKE 3 (DCL3) [102,103,104] (Figure 2a). HUA-ENHANCER 1 (HEN1) methylation modification prevents the degradation of sRNA [105]. Mature sRNA binds to ARGONAUTE 4 (AGO4) [100,106,107] or AGO6 [108,109] and pairs with scaffold RNA transcribed by POL V to recruit DOMAINS REARRANGED METHYLASE 2 (DRM2) [110] for methylation modification (Figure 2c). In addition to the typical POL IV pathway, POL II can also generate siRNA to trigger non-canonical RdDM. While nearly all 24 nt sRNAs involved in RdDM are produced through the POL IV-RDR2-DCL3 pathway, a small proportion are produced through other pathways. For example, some POL II transcripts that contain an inverted repeat sequence form double-stranded hairpin structures that can be directly cleaved by DCL3 to form 24 nt sRNAs [111,112]. For the reverse activation of siRNA genes and some transcriptionally active transposon regions, RdDM relies on POL II and RNA-DEPENDENT RNA POLYMERASE 6 (RDR6) rather than POL IV and RDR2 [108,113,114]. There are also many factors involved in the RdDM passage, such as: AGO4 and/or AGO6 directly associate with POL V, and the association is enhanced by RNA-DIRECTED DNA METHYLATION 3 (RDM3). The production of scaffold RNAs by POL V requires the DDR complex, consisting of the chromatin remodeller DEFECTIVE IN RNA-DIRECTED DNA METHYLATION 1, DEFECTIVE IN MERISTEM SILENCING 3 and RDM1, which associates with both AGO4 and DRM2 and may bind single-stranded methylated DNA [115]. The DDR complex interacts with SUPPRESSOR OF VARIEGATION 3-9 HOMOLOGUE PROTEIN 2 (SUVH2) and SUVH9, which bind to pre-existing methylated cytosines and can recruit POL V [116]. The retention of nascent POL V-transcribed RNA on the chromatin may be facilitated by the RNA-binding proteins RRP6-LIKE 1 (RRP6L1) [117] and the INVOLVED IN DE NOVO 2 (IDN2)–IDN2 PARALOGUE (IDP) complex, which interacts with a SWITCH/SUCROSE NONFERMENTING (SWI/SNF) chromatin-remodelling complex [118,119].

Maintenance methylation refers to the methylation modification performed at the corresponding positions of the nascent strand generated by the semi-conservative replication of methylated DNA. The nascent strand is only methylated at the base positions identical to the methylated positions of the parent strand.

METHYLTRANSFERASE 1 (MET1) was the first plant methyltransferase to be identified and maintains CG methylation in gene-coding regions [120]. In rice, CG methylation is primarily maintained by the *MET1* genes, including *OsMET1a* (*OsMET1-1*) and *OsMET1b* (*OsMET1-2*). Of these genes, *OsMET1b* is the primary methyltransferase responsible for maintaining CG methylation, and its expression is more widespread and at a higher level compared with that of *OsMET1a* [121].

**Figure 3:**
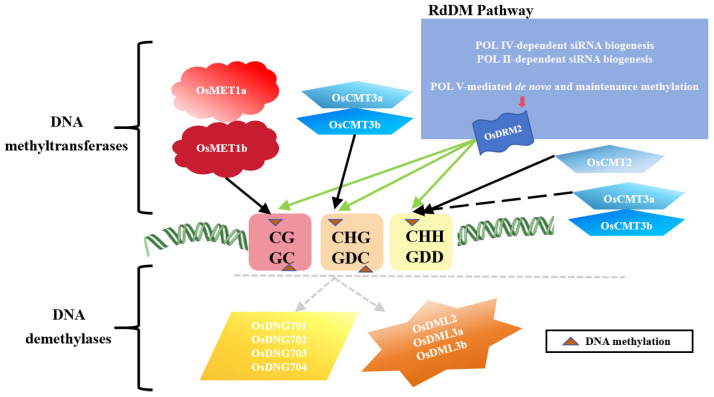
Specific DNA methyltransferases and demethylases mediate cytosine methylation in different sequence contexts.

In plants, non-CG methylation plays a crucial role in regulating gene expression together with CG methylation. CHROMOMETHYLASE 3 (CMT3) and CHROMOMETHYLASE 2 (CMT2) are the plant-specific methyltransferases. The maintenance of CHG methylation mainly depends on catalysis by CMT3, although CMT2 is also involved to some extent [122,123]. Specific DNA methyltransferases and demethylases mediate cytosine methylation in different sequence contexts (Figure 3), loss-of-function of CMT3 and the histone methyltransferase SUVH4 (Su(var)3-9 homolog 4; KRYPTONITE, KYP), which is responsible for H3K9 dimethylation, lead to a significant decrease in DNA methylation levels [124]. Multiple DNA methylation regulatory pathways collaborate to maintain the dense DNA methylation status of jumping genes at different genomic positions. Previously, a “static” model was widely accepted. This model proposed that DNA methylation of a given jumping gene in the genome is maintained by specific pathways. For instance, CHH methylation in the middle of longer jumping genes is maintained by CMT2, while CHH methylation in the edge parts and short jumping genes is maintained by the RdDM pathway. However, more recent studies have proposed a “double insurance” hypothesis: When the function of the RdDM pathway is lacking, the chromatin remodeling factor DDM1-dependent DNA methylation maintenance pathway ensures that jumping genes remain silenced; and when DECREASE IN DNA METHYLATION 1 (DDM1) function is lacking, the RdDM pathway, including the newly discovered CMT2-to-RdDM pathway, inhibits the expression of jumping genes [125].

Methylated CHG recruits the histone H3 lysine 9 (H3K9)-specific methyltransferases SUPPRESSOR OF VARIEGATION 3-9 HOMOLOGUE PROTEIN 4 (SUVH4), SUVH5, and SUVH6. Dimethylated H3K9 (H3K9me2) supports CMT3 and CMT2 activities, creating a reinforcing loop between CHG methylation and H3K9 methylation [123,126,127,128]. DRM2 and CMT2 jointly maintain CHH methylation, with DRM2 regulating CHH methylation in RdDM target regions through the RdDM pathway [110,129]. The rice chromatin methyltransferase *OsCMT3a* maintains the methylation of non-CG (primarily CHG) sites. Mutants of *Oscmt3a* exhibit a significant reduction in CHG methylation, loss of inhibition of transposable elements (TEs) and many genes, and induction of pleiotropic developmental phenotypes that lead to changes in the expression of some genes and TEs, as well as various abnormal developmental phenotypes [130]. Compared with Arabidopsis (*Arabidopsis thaliana* L.), the loss of non-CG methylation in rice causes more obvious growth and development defects than those in Arabidopsis [131]. *OsCMT3* and *OsDRM2* play more important roles in non-CG methylation and development in rice [132].

In contrast to the Arabidopsis genome, the rice genome has a higher CG content, and the CG content decreases from the 5′ to 3′ end of a gene. Given that DNA methyltransferases function in a sequence-dependent manner, a high CG content may increase the level of cytosine methylation [110]. Importantly, the rice genome contains a higher proportion of heterochromatic regions marked by discontinuously distributed TEs; therefore, DNA methyltransferases have a stronger impact on rice development [133]. Compared with *A. thaliana*, this may be due to the methylation of a greater number of TEs and repetitive sequences in rice, which affects the expression of adjacent genes and duplicated genes.

In addition to methylases, DECREASE IN DNA METHYLATION 1 (DDM1), a SWI/SNF chromatin remodeling factor, is essential for maintaining cytosine methylation in genomic repeats and TEs, thus participating in the maintenance of TE silencing in the rice genome. DDM1 does not methylate protein-coding genes, and the mechanism of TE silencing by DDM1 is unique and evolved independent of other silencing pathways without relying on siRNA to enforce the heterochromatic state of TEs [134]. In rice, two genes are homolog orthologous genes to Arabidopsis *DDM1*, namely, *OsDDM1a* and *OsDDM1b* [135]. *OsDDM1* is involved in the maintenance of CG and CHG methylation in heterochromatic and euchromatic regions, as well as CHH methylation in euchromatic regions; however, it suppresses CHH methylation in centromeric repeat sequences [136].

DNA methylation is a reversible epigenetic modification. DNA demethylation in plant genomes can activate silenced genes and can be categorized as passive and active demethylation. Passive DNA demethylation relies on semi-conservative replication of DNA. This process occurs when the activity of DNA methyltransferases is inhibited, or their concentration is low, leading to the replacement of methylated cytosines with unmethylated cytosines and, consequently, a decrease in DNA methylation levels. Active demethylation, in contrast, involves specific enzymatic reactions mediated by DNA glycosylases/lyases. In plant genomes, 5mC can be excised by the ROS1 family of DNA glycosylases/lyases, followed by the synthesis of unmethylated cytosine through base repair mechanisms, resulting in DNA demethylation of the genome [137]. Typically, DNA demethylation is catalyzed by DNA glycosylases, such as REPRESSOR OF SILENCING 1 (ROS1), which prevents hypermethylation of endogenous and transgene loci. DEMETER (DME) plays a crucial role in endosperm genome imprinting. DEMETER-LIKE 2 (DML2) and DML3 are responsible for removing misplaced methylation [138]. The rice genome encodes six recognized 5mC glycosylases, including four enzymes homologous to ROS1 (ROS1a, 1b, 1c, and 1d) and two enzymes homologous to DML3 (DML3a and DML3b) [139,140,141].

### 3.1. DNA Methylation in Response to Drought Stress

Drought stress occurs frequently in the growth cycle of plants. Rice, maize (*Zea mays* L.), wheat (*Triticum aestivum* L.), tomato (*Lycopersicon esculentum*), Arabidopsis, and other plant species have been shown to significantly alter genome-wide DNA methylation under drought stress, with a large number of drought-tolerant genes regulated by DNA methylation.

Compared with other plants, studies on DNA methylation under drought stress in rice are more comprehensive and in-depth. Drought stress causes genome-wide alterations in DNA methylation in rice, accounting for 12% of the total specific sites, and 70% of the DNA methylation sites were restored after the resumption of watering [142]. In another group of studies, the changes in DNA methylation in rice under drought stress were diametrically opposed in different developmental periods, with a significant increase in methylation during the nutritional growth period and a significant decrease in methylation during the reproductive growth period [143]. In addition, a significant genetic effect of DNA methylation in rice under multiple generations of drought stress treatment has been reported [144]. A high percentage of drought-induced epigenetic mutations maintain their modified DNA methylation state. It is thus speculated that DNA methylation may have had a positive effect on the domestication of rice [145]. An overview of the mechanisms of DNA methylation in rice shows that it generally affects the up-(down)regulation of gene expression by acting on the promoter of the gene [18]. Small RNA abundance is positively correlated with hypermethylated regions and interplay exists among DNA methylation, gene expression, and small RNA abundance [146]. DNA methylation is involved in short-term drought memory in rice together with long non-coding RNA (lncRNA) and endogenous phytohormones (especially ABA) [147]. With regard to specific gene functions, *cld1* controls leaf curling in rice by affecting bulliform cell formation, whereas DNA methylation can deactivate it and influence drought tolerance [148].

In addition to rice, many studies of DNA methylation and drought stress have been undertaken in other plant species. In many species, a relationship between methylation and drought stress is observed. In maize, hundreds of differentially expressed DNA methylation-associated 24 nt siRNA clusters overlap with differentially expressed genes in maize treated with rehydration after drought stress [149]. At the individual gene level, an 82 bp TE repressed *ZmNAC111* expression by methylation, thereby altering the drought tolerance of maize [150]. Drought stress caused a significant 10% increase in genome-wide methylation in wheat, and the degree of methylation showed significant tissue specificity [151]. Wheat GLYCERALDEHYDE-3-PHOSPHATE DEHYDROGENASE C SUBUNIT 1 (*TaGAPC1*), owing to differences in DNA methylation levels in its promoter, confers different degrees of drought stress tolerance in different wheat varieties [152]. In tomatoes, approximately 75 methylation tags in *Asr1* gene sequences were removed from 110 CHH methylation sites under drought stress [153]. However, in the model plant Arabidopsis, drought stress only slightly alters genome-wide methylation, and mild drought stress does not induce transgenerational epigenetic effects [154]. In rice, cultivar-specific DNA methylation patterns are potentially an important regulatory mechanism for sensing and response to drought stress via modulation of stress-responsive gene expression. Many of the genes are known to be abiotic stress-responsive [155]. Garg et al. [146] showed that in different drought-tolerant/intolerant varieties of rice, genes encoding transcription factors (of the MYB, AP2-EREBP, WRKY, NAC, and HB families), sodium transporter HKT1 homologs, F-box, calcium-dependent protein kinases, proteinases, peptidases, oxidoreductase, glutathione *S*-transferase, histone deacetylase, and putative dicer-like proteins are differentially methylated. Waseem et al. [156] observed different genome-wide methylation levels among members of the cytochrome P450 eukaryotic gene superfamily in rice. Thus, the effects of DNA methylation vary widely among crops and the mechanisms of interactions require more detailed investigation.

DNA methylation in response to drought stress in rice is distinctly species-, genome-, tissue-, and period-specific. With growth in the body of research, an increasing number of correlations between drought stress and DNA methylation have been reported. However, further refinement of the specific mechanism of association is required.

### 3.2. DNA Methylation in Response to Temperature Stress

Temperature stress often occurs during the entire growth cycle of plants. Freezing or low temperatures are critical factors that affect plant growth, development, and crop yield. Heat stress is a serious threat to the growth and development of crops worldwide and leads to a series of morphological, physiological, and biochemical changes in plants. Almost all organisms have evolved signaling pathways to sense changes in environmental temperature. DNA methylation changes are an important means for plants to regulate gene expression in response to temperature stress [157].

On the basis of the results of fluorescence in situ hybridization with 45S and 5S rDNA and centromeric probes, in rice heat stresses cause extensive decondensation of 45S rDNA chromatin and an increase in the distance between the two homologous 5S rDNA loci. The DNA hypomethylating agent, 5-azacytidine, significantly enhances the decondensation of 45S rDNA chromatin and, interestingly, exhibits the capacity to trigger the polarization of centromeres within interphase nuclei [158]. Under low-temperature stress in Arabidopsis, DNA demethylation occurs in the promoter regions of *ACD6*, *ACO3*, and *GSTF14* in the RdDM pathway. This demethylation leads to reduced expression of these genes while simultaneously activating the expression of genes related to defense and stress resistance. ROS1-mediated DNA demethylation plays a crucial role in this process [159]. In tea plants, analyses of DNA methylation at single-base resolution and gene expression profiles under low-temperature stress revealed that CG methylation is negatively correlated with gene expression, whereas CHG and CHH methylation in promoter regions are positively correlated with gene expression. Low temperatures induce the downregulation of genes encoding DNA methyltransferases and upregulation of genes encoding demethylases in tea plants. The demethylation of key cold-responsive genes, such as *CBF4,* contributes to the cold stress response [160].

In the rice cold-tolerant cultivar P427, 51 genes show concurrent changes in methylation and expression levels under cold stress. In addition, genes involved in the INDUCER OF CBF EXPRESSION–C REPEAT BINDING FACTOR–COLD REGULATION (ICE–CBF–COR) pathway are highly expressed under cold stress. The mechanism of ICE–CBF–COR signaling is mediated through the promoter of the rice *OPEN STOMATA 1* (*OST1*) ortholog (*Os03g0610900*), which may interact with and phosphorylate ICE1 and increase its expression level. Cold stress treatment significantly increases expression of *HIGH EXPRESSION OF THE OSMOTICALLY RESPONSIVE GENES 1* (*OsHOS1* and *Os03g0737200*) and *ETHYLENE RESPONSE FACTOR 20* (*ERF20*, *OsDREB1G,* and *Os02g0677300*) in P427, indicating that methylation of the cold-dependent *OST1* in P427 mediates the ICE–CBF–COR cold signal transduction pathway [161]. The thermal sensitivity of rice seed size may be caused by changes in the epigenetic regulation of endosperm development. The expression of rice *FERTILIZATION-INDEPENDENT ENDOSPERM 1* (*OsFIE1*) is temperature-sensitive. An investigation of the molecular mechanisms underlying rice thermosensitivity and seed size reduction controlled by *OsFIE1* indicated that altered DNA methylation and histone methylation (H3K9me2) may be the main factors regulating *OsFIE* [21]. In addition to rice, DNA methylation and temperature stress have been studied in other plant species. Both methylation and demethylation occur during cold adaptation. In *Brassica*, a total of 1562 differentially methylated genes have been identified during cold adaptation, including MITOCHONDRIAL MALATE DEHYDROGENASE 1 (BramMDH1), 3-KETOACYL-COA THIOLASE-2 (BraKAT2), SERINE HYDROXYMETHYLTRANSFERASE 4 (BraSHM4), and 4-COUMARATE COENZYME A LIGASE 2 (Bra4CL2), whose promoters were demethylated and led to increased transcriptional activity [162]. In cotton, high temperatures cause global disruption of DNA methylation, particularly affecting CHH methylation in the anthers. Inhibition of DNA methylation results in pollen sterility in heat-sensitive cotton varieties [163].

### 3.3. DNA Methylation in Response to Salt Stress

Salt stress reduces land and water productivity and exacerbates poverty and food insecurity. The increase in salinization caused by human practices and climate change is gradually reducing agricultural productivity [164].

Increasing evidence suggests that DNA methylation plays an important role in regulating gene expression in response to salinity [165]. Epigenetic modification of salt stress response genes can modulate the response to salt stress in rice. Tissue-specific DNA methylation changes induced by salinity stress have been observed in rice. In root tissues, salt stress significantly reduced the methylation levels, but only minor changes in the methylation levels were observed in leaf tissues [166]. In addition to tissue specificity, there are also significant differences in their varieties. Hypermethylation has been observed in salt stress-tolerant rice genotypes, whereas salt stress-sensitive genotypes showed demethylation [167]. Salt-sensitive rice cultivars had a greater abundance of salt-induced transcripts in buds than salt-tolerant rice cultivars compared with leaves [168]. The expression of ABSCISIC ACID RESPONSE ELEMENT BINDING FACTOR 8 (OsBZ8) was more highly induced in the salt-tolerant rice cultivar Nonabokra compared with that of IR64, and the absence of DNA methylation was observed in OsBZ8 [169].

DNA methylation and salt stress have been investigated in plant species other than rice. The DNA methylation changes caused by the somatic cell hybridization of the salt-tolerant wheat line (SR3) may be partly responsible for the enhanced salt tolerance [170]. RNA-DIRECTED DNA METHYLATION 16 (RDM16) is a factor in the RdDM pathway that regulates DNA methylation by affecting the transcriptional levels of POL V to enhance the ability of plants to cope with salt stress [171]. In plants under salt stress, DNA methyltransferase, and domain-rearranged methyltransferases induce DNA methylation variation and bring about plasticity, which in turn affects the expression levels of some members of the CMT and MET [172].

### 3.4. DNA Methylation in Response to Heavy Metal Stress

Heavy metal stress has less impact on crop yield than other abiotic stresses. However, the strong capacity for heavy metal transfer in the food chain can have a serious impact on humans. With the increase in heavy metal contamination of agricultural soils, the correlation between genomic DNA methylation and heavy metal stress in plants has been increasingly studied. In particular, many similar studies have been undertaken in rice, maize, wheat, soybean (*Glycine max* (L.) Merr.), Arabidopsis, rapeseed, and other species. Among all heavy metals, Cd stress has been studied the most intensively because it has the greatest impact [173].

Correlation analysis of various heavy metal stresses and DNA methylation has been extensively studied in rice. Many differential DNA methylation marker sites have been detected in rice under different Cd stress environments, and 2320 non-redundant differentially methylated regions were detected in the genome. The expression of many genes modified by methylation is altered significantly, with a greater proportion of genes being hypermethylated [174]. Methylation patterns differ among rice genotypes subjected to heavy metal stress, and the strategies to confer tolerance also differ among genotypes. By correlating the available results with gene expression changes, a strong relationship between heavy metal transport genes and DNA methylation patterns has been observed [175]. By measuring the DNA methylation pattern of rice under heavy metal stress in different generations, it was observed that the methylation status of the heavy metal-transporting P-type ATPase-related retrotransposon Tos17 showed cross-generational inheritance [176]. In addition to specific genes, the progeny of stressed plants exhibit enhanced tolerance to the same stresses experienced by their progenitors, and this transgenic inheritance is accompanied by the heritability of modified methylation patterns [177]. In specific gene function studies, overexpression of the metal detoxification transporter protein *OsZIP1* confers superior growth and reduces heavy metal accumulation in rice exposed to heavy metal stress. The characterization of the DNA methylation of the *OsZIP1* histone H3K9me2 revealed that its transcribed regional sites were demethylated [178]. The overexpression of the gene encoding the heavy metal response protein OsHMP enhances the growth of rice under Cd stress. Unlike *OsZIP1*, overexpression of *OsHMP* results in enhanced accumulation of Cd, but the same pattern of DNA methylation and demethylation led to upregulation of the gene [179].

As a comparison with data available for rice, DNA methylation, and heavy metal stress in maize, wheat, horsebean (*Vicia faba* L.), radish (*Raphanus sativus* L.), and Arabidopsis are considered here. In maize, heavy metal stress causes genome-wide DNA methylation changes. The available results show that 3857 differentially methylated genes were identified in four groups of maize samples under Pb stress, and these genes encoded numerous phytohormone-related transcription factors [180]. In addition to Pb, Zn causes significant changes in DNA methylation patterns in maize and affects the promoters of a large number of genes, resulting in altered gene expression [181]. In contrast, in wheat, DNA methylation induced by Pb and Cd is clearly associated with metal detoxification transporter proteins [182]. In the model species *Arabidopsis thaliana*, Cd stress increases genome-wide DNA methylation through inhibition of demethylases to improve plant stress tolerance [183]. Among multiple Arabidopsis populations subjected to Cd treatment, a population with a higher DNA methylation level exhibited greater Cd tolerance [184].

**Figure 4:**
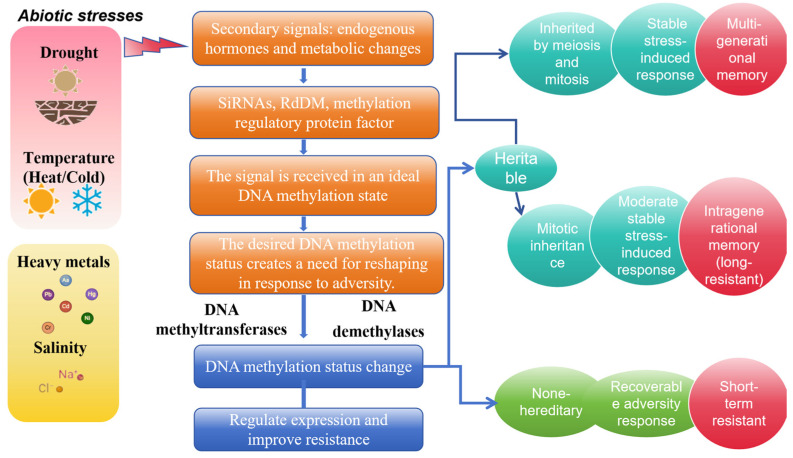
Schematic illustration of DNA methylation and abiotic stress tolerance in rice.

Based on the foregoing literature, it is concluded that heavy metal stress can also cause DNA methylation alterations in diverse plant genomes. With respect to epigenetic continuity, heavy metal stress appears to have a greater and more pronounced effect than drought stress (Figure 4).

## 4. Discussion and Future Prospects

Abiotic stress is a significant factor limiting global crop yield. Drought, temperature, salinity, heavy metals, and other abiotic stresses impose substantial negative impacts on the overall growth and development of plants, particularly during reproductive stages. While considerable progress has been made in identifying and functionally analyzing genes related to abiotic stress in crops, most stress-resistant genes remain impractical for direct application. First, plant tolerance and yield are often antagonistic; most stress-resistant genes directly lead to reduced crop yield under normal conditions. Second, genomic changes affect every stage of crop growth, failing to induce crop-specific responses to external stresses, which often occur in stages and specificity. Therefore, epigenetic changes (DNA methylation) can compensate for the shortcomings of direct genomic alterations or overexpression of target genes, influencing gene expression regulation without affecting genomic changes. This review summarizes genes in rice responsive to abiotic stress and related information (Table 1), as well as functional genes undergoing DNA methylation changes under abiotic stress (Table 2), as discussed in the literature. Changes in DNA methylation typically affect gene expression, and in plants, DNA methylation of gene promoters is generally associated with gene silencing [185]. Among various types of DNA methylation, mCG methylation around transcription start sites is most strongly linked to gene silencing [186]. For effective silencing, promoter methylation requires additional effectors, such as methyl-DNA binding proteins, histone modifiers, chromatin remodeling factors, and molecular chaperones. These proteins function together to form a compact chromatin structure that transcription factors cannot access [187,188]. Beyond gene silencing, integrated analyses of methylomes and transcriptomes in multiple plant species have revealed a positive correlation between high promoter methylation levels and the expression of specific subsets of genes [189]. These findings suggest that DNA methylation in certain genomic regions, such as promoters, does not solely function to silence or activate gene expression. Instead, it is crucial to focus on specific gene base sequences or corresponding three-dimensional conformations to determine how DNA methylation influences gene expression levels through more detailed structural comparisons.

Further research on DNA methylation and crop stress resistance should focus on several key areas. First, comprehensive DNA methylation sequencing of model crops at the population level, combined with single-cell methylation analysis in various tissues, can provide detailed insights. Integrating macro- and micro-level data and supplementing with multi-omics data from different developmental stages and environmental conditions will help to build a gene regulatory network at the multi-omics level. This approach will identify crucial DNA methylation sites and regions associated with stress resistance, enhancing our understanding of epigenetic mechanisms. Additionally, the use of advanced techniques such as machine learning and cryo-electron microscopy will provide new information about the mechanisms and structural changes in key methylation sites identified through omics data. Using CRISPR/Cas9 for precise modification of DNA methyltransferase and demethylase genes, as well as stress-responsive methylation regions, will enable targeted changes in gene methylation status to regulate gene expression. Finally, identifying epigenetic markers associated with crop tolerance through genome-wide methylation sequencing will facilitate crop breeding, either by transferring beneficial methylation states to subsequent generations or by precisely editing key methylation sites in genes related to stress resistance.

## Figures and Tables

**Table 1:** Genes in rice responding to abiotic stress.

Gene Name	Annotation	Locus ID	Reference		Function
*OsMYBR1*	MYB Transcription factor	LOC_Os04g49450	[36]	Drought Stress	OsMYBR1-RNAi with higher than WT survival after restored watering, lower relative conductivity and malondialdehyde content, and higher proline content, which negatively regulated drought tolerance in rice
*OsMYB48; OsMYB48-1*	MYB Transcription factor	LOC_Os01g74410	[37]	Drought Stress	OE-OsMYB48-1 showed reduced water loss rate, low malondialdehyde, and high proline, hypersensitivity to ABA during germination and after germination, and accumulated more endogenous ABA under drought stress.
*OsPIN3t; OsPIN10a; OsPIN3a*	PIN-FORMED	LOC_Os01g45550	[39]	Drought Stress	OsPIN3t Is an auxin export carrier localized to the plasma membrane, involved in the polar auxin transport, but also involved in the drought stress response in rice
*OsGH3-2; OsGH3.2*	indole-3-acetic acid-amido synthetase	LOC_Os01g55940	[40]	Drought Stress	OsGH 3-2 encodes an enzyme that catalyzes IAA-binding amino acids, which is induced by drought but inhibited by cooling. It is involved in the regulation of auxin and abscisic acid content in rice and plays positive and negative roles in regulating cold tolerance and drought resistance, respectively
*SRL1; CLD1*	SEMI-ROLLED LEAF1	LOC_Os07g01240	[129]	Drought Stress	SEMI-ROLLED LEAF1 encodes a glycosylphosphatidylinositol-anchored protein localized to the plasma membrane. SRL 1 negatively regulates vesicular cell formation by negatively regulating the expression of the vacuolar H + -ATPase subunit and H + -pyrophosphatase genes
*OsNAC10; ONAC122(ZmNAC111)*	NAC Transcription factor	LOC_Os11g03300	[131]	Drought Stress	OsNAC10 The specific expression in rice roots increases the root system, enhances the drought resistance ability, and then improves the rice yield under drought conditions
*OsSAPK10*	Stress-Activated Protein Kinase	LOC_Os03g41460	[139]	Drought Stress	miR2105 and the kinase OsSAPK10 co-regulate OsbZIP86 to mediate drought-induced ABA biosynthesis in rice.
*OsNRAMP5*	natural resistance-associated macrophage protein	LOC_Os07g15370	[73]	Heavy Metal	Nramp5 is a resistance-related macrophage protein, the main transporter of rice root cells involved in the uptake of external metal, and is also responsible for the transport of these ions from the roots to the shoots.
*OsIRT1*	iron-regulated transporter	LOC_Os03g46470	[73]	Heavy Metal	OE-OsIRT1 showing increased resistance to iron deficiency at the seedling stage, sensitivity to excess zinc and cadmium, and increased zinc–iron content in shoots, roots, and mature seeds.
*OsIRT2*	iron-regulated transporter	LOC_Os03g46454	[73]	Heavy Metal	Rice plants can absorb cadmium ions from the soil through OsIRT1 and OsIRT2 and transport them to the ground, and OE-OsIRT2 enhances cadmium stress resistance.
*COLD1*	Chilling Tolerance	LOC_Os04g51180	[53]	Temperature Stress	COLD 1 encodes a regulator of G protein signaling, and overexpression of COLD1jap significantly increases rice cold tolerance, while rice lines lacking or low expression of COLD1jap are sensitive to cold.
*D1*; *RGA1*; *D89*	heterotrimeric G protein α subunit	LOC_Os05g26890	[53]	Temperature Stress	When experiments at the same light intensity, d1 showed a stronger ability to eliminate excess irradiance with increased non-photochemical quenching. Increased light avoidance and photoprotection in d1 reduced sustained photoinhibitory damage, as revealed by higher Fv/Fm.
*OsEIL1*	ethylene-insensitive	LOC_Os03g20790	[63]	Salt Stress	MHZ 6 encodes OsEIL1, which is homologous to EIN 3, a major transcriptional regulator of ethylene signaling in Arabidopsis, with transcriptional activation activity. MHZ 6/OsEIL1 and OsEIL2 negatively regulate salt tolerance in rice, which may be achieved through direct regulation of OsHKT2; 1 expression and Na + uptake in roots.
*OsEIL2*	ethylene-insensitive	LOC_Os07g48630	[63]	Salt Stress	OsEIL2 Loss of function will improve salt tolerance, with less Na + accumulation in root and shoots under salt stress; the seedlings of overexpression lines are sensitive to salt, with more Na + accumulation and lower grain size and 1000-grain weight.
*OSBZ8*; *OsbZIP05*; *OsbZIP5*	Bzip Transcription factor	LOC_Os01g46970	[151]	Salt Stress	OSBZ 8 plays an important role in the transcriptional regulation of vegetative tissues in rice. OSBZ 8 is present in the ABRE-DNA: protein complex, and when treating seedlings with NaCl increases complex formation. OSBZ 8 is regulated at both the transcriptional and post-transcriptional levels. There is a positive correlation between OSBZ8 expression and salt resistance.

**Table 2:** Target genes affected by DNA methylation in rice under abiotic stress.

Gene Name	Annotation	Locus ID	Reference		Function
*ZFP31*	LSD0 subclass family protein	LOC_Os07g17400	[190]	Drought Stress	zinc finger, RING-type, The methylation content of gene ZFP31 decreased, and the expression level increased under salt stress
*ZP160*	LSD1 subclass family protein	LOC_Os08g12680	[190]	Drought Stress	zinc finger domain, LSD1 subclass family protein The methylation content of gene ZP160 decreased, and the expression level increased under salt stress
*ZP35*	ZOS11-03-C2H2 zinc finger protein	LOC_Os11g30484	[190]	Drought Stress	zinc finger domain, LSD2 subclass family protein The ZFP35 gene was downregulated, and methylation was reduced under salt stress and drought stress
*OsTRAB1;OsbZIP66*	bZIP Transcription factors	LOC_Os08g36790	[146]	Drought Stress	Enhances demethylation and increases expression level under drought stress. Hypomethylation was mainly observed in the flanking region (88.88%)
*OsRHP1*	RING-H2 finger protein	LOC_Os08g38460	[146]	Drought Stress	
*OsAP2.4*	AP2 domain-containing protein expressed	LOC_Os04g57340	[146]	Drought Stress	
*OsSGL;An-4*	SOG1-like	LOC_Os02g38130	[146]	Drought Stress	
*OsDOG1L-1*	SOG1-like	LOC_Os01g06560	[146]	Drought Stress	
*OsGDA1*	guanine deaminase 1	LOC_Os03g61810	[191]	Drought Stress	OsGDA1 Knockdown Impacts Xanthine Metabolism and SAH Content. Lower SAH can enhance genomic methylation, altering gene silencing or expression.
*DUF3353*	DUF family protein	LOC_Os03g15033	[192]	Drought Stress	DUF3353 is downregulated in drought stress and is targeted by 20 miRNAs that are part of miRBase
*EL268*	Snf2	LOC_Os03g51020	[193]	Drought Stress	Differential methylation and regulation of Snf 2 family protein genes may lead to epitopes differentially methylated genome-wide when rice is subjected to osmotic (drought) stress
*OsZIP1*	zinc-regulated transporters and iron-regulated transporter-like protein	LOC_Os01g74110	[142]	Heavy Metal	OsZIP1 Is a metal detoxification transporter that prevents excessive accumulation of zinc, copper, and cadmium in rice. The DNA methylation of OsZIP1 histone H3K9me2 was further characterized by finding that its transcribed regional sites were demethylated
*OsHMP*	Heavy Metal Responsive Protein	LOC_Os02g37280	[143]	Heavy Metal	heavy metal transport/detoxification protein in rice
*Oshox22*	Homeobox-leucine zipper protein HOX1	LOC_Os04g45810	[146]	Salt Stress	In Pokkali, the CHH background showed hypermethylation and higher gene expression under salinity stress
*Oschit1.1*	glycosyl hydrolase, putative, expressed	LOC_Os01g64100	[146]	Salt Stress	In Pokkali, the CHH background showed hypermethylation and higher gene expression under salinity stress
*OSBZ8;OsbZIP05*	bZIP Transcription factors	LOC_Os01g46970	[169]	Salt Stress	The DNA hypomethylation status at the OsBZ 8 locus may promote transcript expression of salt tolerance genes.
*OsBAG4*	BAG protein	LOC_Os01g61500	[169]	Salt Stress	Enhances resistance to salt stress by protein complex OsSUVH7-OsBAG4-OsMYB106, and thus binding OsHKT1, regulates NA + to achieve.
*OsSUVH7;SDG709*	DNA methylation reader	LOC_Os01g59620	[169]	Salt Stress	
*OsMYB106*	MYB Transcription factors	LOC_Os08g33660	[169]	Salt Stress	
*OsHKT1;5*	Na+-selective transporter	LOC_Os01g20160	[169]	Salt Stress	
*OsMET1-2*	DNA methyltransferase	LOC_Os07g08500	[194]	Salt Stress	Suppresses expression under salt and drought stress
*OsCMT2*	chromomethylase	LOC_Os05g13780	[194]	Salt Stress	Enhances resistance to cold and salt stress
*OsCMT3*	chromomethylase	LOC_Os10g01570/LOC_Os03g12570	[194]	Salt Stress	Suppresses expression under salt and drought stress as DNA methylase
*TIR1*	auxin receptor	LOC_Os05g05800	[195]	Salt Stress	Rice produced salinity adaptation by the methylation differences in the promoter region of the osa-miR393a-TIR 1 module.
*OST1*	Stress-Activated Protein Kinase	LOC_Os03g41460	[196]	Temperature Stress	Interacts with and phosphorylates ICE1 to avoid ubiquitination degradation of the ICE1 protein, thereby enhancing the plant’s ability to tolerate chilling
*PPR*	PPR repeat domain-containing protein, putative, expressed	LOC_Os07g28900	[197]	Temperature Stress	Cytosine methylations in the promoter regions of genes involved in the cellular oxidation equilibrium pathways affect rice heat tolerance
*OsFIE1*	fertilization-independent endosperm gene	LOC_Os08g04290	[21]	Temperature Stress	Rice fertilization-independentendosperm1 regulates seed size under heat stress by controlling early endosperm development.

## Data Availability

Not applicable.
